# Supplementary material for: High Heritabilities for Antibiotic Usage Show Potential to Breed for Disease Resistance in Finishing Pigs
Source: Antibiotics (Basel). 2021 Jul 8;10(7):829. doi: 10.3390/antibiotics10070829 (PMC8300637; doi:10.3390/antibiotics10070829)
Supplement: Supplementary file 1 [file antibiotics-10-00829-s001.zip › antibiotics-1279160-supplementary.pdf]

Table S1. Extra information on administered antibiotics

Additional File 1 Table S1. Extra information on administered antibiotics. The LA-factor (Long Acting) is used to calculate treatment incidences to account for a possible long acting effect (AMCRA, 2020). *Only parenterally administered antibiotics were used in this study.*

Class antibiotics	Active compound	Commercial drug name	Concentration (mg/ml)	LA-factor
Amfenicoles	Florfenicol	Florfenikel®, Kela	300	2
Aminopenicillines	Amoxicillin	Vetrimoxin Long Acting®, Ceva Santé Animale NV	150	1
Aminosides	Paromomycin	Gabbrovet®, Ceva Santé Animale NV	175	1
Cephalosporins	Ceftiofur	Ceftiosan®, Alfasan International BV	50	1
Cephalosporins	Cefquinome	Cobactan 2,5%®, Intervet International BV	25	1
Cephalosporins	Ceftiofur	Excenel Flow®, Zoetis Belgium SA	50	1
Fluoroquinolones	Enrofloxacin	Fenoflox®, Chanelle Pharmaceuticals Manufacturing Ltd	50	1
Lincosamides	Lincomycin	Lincomycine-VMD®, VMD NV	100	1
Macrolides	Tulathromycin	Draxxin®, Zoetis Belgium SA	100	9
sulfonamides-trimethoprim	Trimethoprim<br>Sulfadoxine	Dofatrim-ject®, Dopharma Research BV	40<br>200	1
